# Pyrometallurgical Approach to Extracting Valuable Metals from a Combination of Diverse Li-Ion Batteries’ Black Mass

**DOI:** 10.1021/acssusresmgt.4c00117

**Published:** 2024-07-22

**Authors:** Safoura Babanejad, Hesham Ahmed, Charlotte Andersson, Elsayed Mousa

**Affiliations:** † Department of Civil, Environmental and Natural Resource Engineering, Process Metallurgy, Minerals and Metallurgical Engineering, Luleå University of Technology, 97187 Luleå, Sweden; ‡ Central Metallurgical Research and Development Institute, P.O. Box 87, Helwan 11421, Egypt; § SWERIM AB, Aronstorpsvägen 1, 97437, Luleå, Sweden

**Keywords:** lithium-ion batteries, black mass from different battery types, pyrometallurgy, alloy, mechanical activation, high-temperature transformation

## Abstract

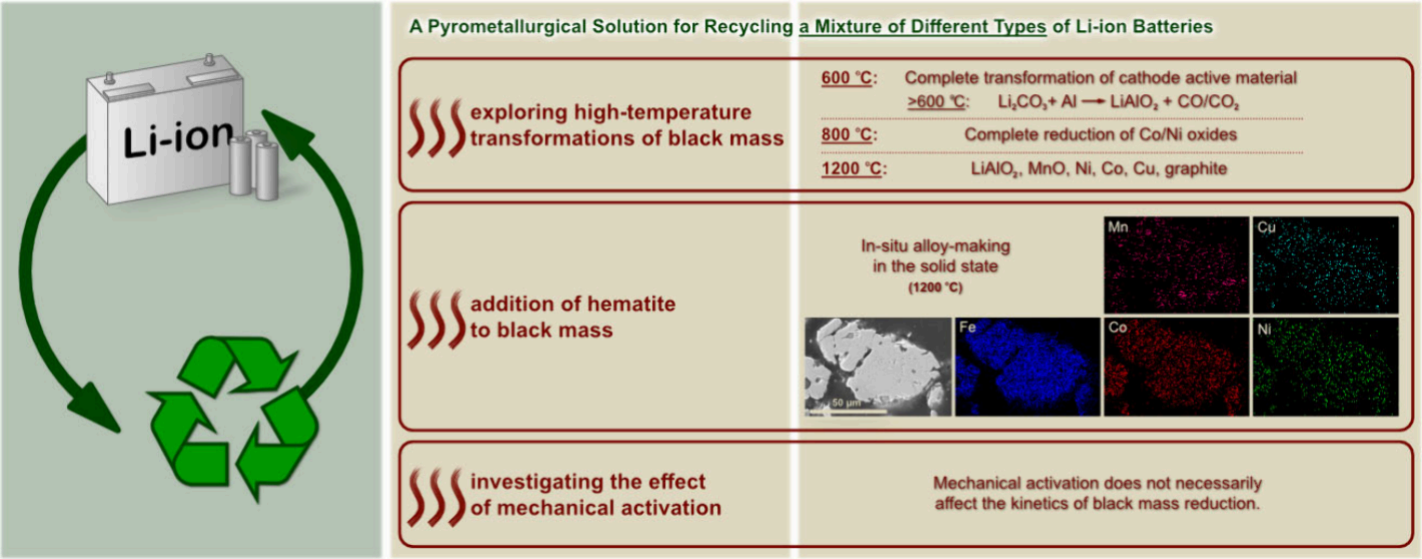

Li-ion batteries (LIBs) are widely used nowadays. Because of their limited lifetimes and resource constraints in manufacturing them, it is essential to develop effective recycling routes to recover their valuable elements. This study focuses on the pyrometallurgical recycling of black mass (BM) from a mixture of different LIBs. In this study, the high-temperature behavior of two types of mixed BM is initially examined. Subsequently, the effect of mechanical activation on the BM reduction kinetics is investigated. Finally, hematite is added to the BM to first be reduced by the excess graphite in the BM and second to form an Fe-based alloy containing Co and Ni. This study demonstrates that mechanical activation does not necessarily affect the kinetics of BM high-temperature behavior. Furthermore, it demonstrates that alloy-making by the addition of hematite is a successful method to simultaneously utilize the graphite in the BM and recover Co and Ni, regardless of the LIB type.

## Abstract 2

When the sustainability in pyrometallurgical recycling of Li-ion batteries was enhanced, precious metals were recovered through an in situ alloy-making process, with an attempt to decrease CO_2_ emissions.

## 1 Introduction

Today, we see a transition from fossil fuels to renewable energies.^1,2^ Rechargeable batteries play a vital role in this transition since the use of renewable energies is not possible without storing them, and rechargeable batteries are necessary for this purpose. Li-ion batteries (LIBs), with their unique properties, are the most popular type of rechargeable batteries.^3,4^ Among their unique properties, one can mention the high energy density, the large charge cycle, and no memory effect.^5^ The anticipated increase in the use of these batteries is likely to result in an increase in discarded LIBs in the upcoming years. Therefore, establishing proper recycling methods is necessary to realize LIBs as resources for valuable products. Achieving this aim will help to control environmental and human health hazards, create economic values, and reclaim material.^6−11^

In the manufacturing of LIBs, approximately 50% of the cost and material is dedicated to the battery cell.^12,13^ Battery cells mainly consist of a cathode, an anode, a separator, and current collectors. Hence, there is significant potential to recover materials from the battery cell components.^14−17^ In the recycling process, after applying some mechanical treatments on these battery cells, a black fine material is obtained, which is known as black mass (BM).^18,19^ BM contains various elements, among which Mn, graphite, Si, Co, Ni, and Li are announced as critical elements by the European Commission.^20^

The recovery process continues to employ pyrometallurgical and/or hydrometallurgical techniques.^7,12,21,22^ Studies of pyrometallurgical techniques fall into two categories. In the first group, pyrometallurgy serves as a pretreatment step for hydrometallurgical techniques, focusing on temperatures between 400 and 800 °C to obtain a material with proper leachability.^23−27^ The second group focuses on pyrometallurgy as a standalone recovery technique, with temperatures higher than 1400 °C, associated with melting the BM and utilizing slag formers.^28−32^ Due to the high amount of graphite in BM, graphite plays a decisive role in selecting the pyrometallurgical process. As an example for the first group, Lombardo studied the pyrolysis and incineration of BM (a mixture of cathode and anode active material—scraped from electrode foils), at a temperature range of 400–700 °C. The product of pyrolysis was a material containing Co, Co oxide, Ni, Ni oxide, Mn, Mn oxide, Li_2_O, Li_2_CO_3_, and C. Despite the presence of C, which inhibits leaching, the produced compounds had a better leachability compared to Li metal oxide (LiMeO_2_) in BM.^33^ Other studies used a similar approach with manually prepared materials, i.e., a mixture of cathode and anode active material [LiCoO_2_ (LCO) and graphite]^34^ and the cathode (LCO and Al foil),^35^ from spent LIBs. Manual separation of LIBs in these studies can yield results different from those obtained with industrial BM. An industrial BM was studied by Vishvakarma and Dhawan, where a BM from a mixture of LIBs was prepared, applying mechanical treatment and different physical separation techniques.^36^ Physical separation applied in that study led to a low Al content in the produced BM, which is not always the case in the industrial processes for BM production. Thus, it can be understood that the resulting product varies significantly depending on the composition of the input BM, particularly in the presence of Al.^24,37−39^ In the second group, the focus is on Li recovery at high temperatures using slag systems.^23,28,29,40−42^ In these studies dealing with melting the BM, the experiments were conducted in air, which obliged incineration of graphite. This phenomenon is also observed in industries like Umicore in Belgium, where LIBs enter the furnace without pretreatment.^43,44^ To address this issue, the addition of metal oxide for the consumption and utilization of graphite was studied.^23,41,42^ In these studies, because of the presence of graphite in the surrounding environment, CuO addition was solely based on observation; i.e., CuO was added as long as graphite was visible on the slag surface. CO_2_ emission is another concern associated with this method. Researchers have suggested a solution to control and reduce CO_2_ emission, which is the application of mechanical activation. Studies have shown that mechanical activation decreases the temperature required for the reduction of oxides, e.g., hematite, ilmenite, and manganese ore,^45−47^ which lowers CO_2_ emission.

Considering the aforementioned works, some of the flaws with the previous works can be pointed out as follows: (i) uncertain explanations for high-temperature transformations in BM; (ii) loss of graphite in the air atmosphere; (iii) observation-based addition of metal oxides for the utilization of graphite. To amend these flaws and strengthen the knowledge in pyrometallurgical recycling of LIBs, the authors of the current work studied first the reduction behavior of BM^48^ and then the addition of metal oxides to BM.^49^ In the first study, the reduction behavior of BM from two types of LIBs (one type at a time) was investigated at elevated temperatures (up to 1100 °C) and the gradual changes in the BM by increasing the temperature were elaborated. In the second study, metal oxides [hematite and Cu(II) oxide] were added to the BM (similar to the BM types in the first study) in an inert atmosphere (with a maximum working temperature of 1450 °C), and the effect of milling on the reduction and alloying processes was investigated. Obtained from that study was that it is feasible to produce an Fe–Cu-based alloy containing Co and Ni as the alloying elements. Additionally, it was observed that increasing the milling time enhances the reaction kinetics, resulting in an increase in the amount of C remaining in the sample. The mass and energy balance of the system in the alloying processes were also studied to support the experimental findings. To extend the study from the laboratory to an industrial scale, another type of BM was used in the current study. In industrial pyrometallurgical practices, different types of LIBs are typically not separated to enter the recycling process, and there is usually a mixture of different LIB types that is being processed.^50^ Thus, instead of using a BM obtained from a single type of LIB, two industrial BMs (produced from a mixture of different LIBs) have been considered, and their high-temperature behaviors have been studied. Subsequently, the effect of milling was taken into account, and in the next step, the feasibility of solid-state alloying (at 1200 °C) was investigated by the addition of hematite. In summary, the goal of this study, compared to previous studies, is to observe the high-temperature behavior of BM from a mixture of LIBs, reassess the impact of milling, and explore the possibility of alloy-making during solid-state reduction.

## 2 Materials and Methods

### 2.1 Materials

In this study, two types of BMs were used [high Co (HiCo) BM and low Co (LoCo) BM], originating from the processing of a mixture of different NMC LIBs (cathode active material: LiNi_*x*_Mn_*y*_Co_1–*x*–*y*_O_2_), which were produced at a recycling facility. Hematite (−325 mesh, 98%, Alfa Aesar) was utilized in addition to BM in the alloying trials.

### 2.2 Methods

A Fritsch Pulverisette 7 Planetary micro ball mill was used to mechanically activate the BM and the mixture of BMs with hematite. The 80 mL cups in this ball mill consist of 10 hardened steel balls with a diameter of 15 mm. To avoid excessive heat during milling, each 30 min of milling was followed by 15 min of cooling. The sample-to-balls ratio and rotating speed were fixed. A total of ∼20 g of sample and 10 balls were rotated at 700 rpm for 30, 60, and 90 min for milling of the BM. The optimum condition was later used for the milling of a hematite and BM mixture.

The unmilled and milled (mechanically activated) BM samples (HiCo BM and LoCo BM) and their mixtures with hematite were separately heated in a Netzsch STA 409 instrument in order to conduct thermogravimetric analysis (TGA). The detection limit was ±1 μg, and the experiments were run under an Ar atmosphere (flow rate of 100 mL/min). The samples were heated from room temperature to 1200 °C at a rate of 10 °C/min and kept at that temperature for 60 min, followed by 20 °C/min cooling. The effect of mechanical activation was investigated by analyzing the materials before and after mechanical activation, as described in the following section. In order to investigate the reactions occurring during heating, differential thermal analysis (DTA) was employed for the unmilled samples in parallel with TGA.

For the alloying trials, hematite was added to the BM with specified ratios. The following reactions (eqs 1 and 2) were supposed as the main reactions in this study, assuming that CO is the product of the reaction of C with O:
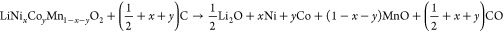
1
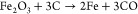
2Thus, the amount of added hematite has been calculated by eq 3 to achieve a total C/O molar ratio of 1:
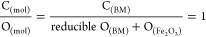
3

### 2.3 Characterization

To analyze the amount of reducible O (at 1070 °C) and C in the BM, a CHNS-O elemental analyzer from Eurovector Srl was utilized, according to the DIN 51732 standard. The ion-selective electron method was also used to analyze the amount of F in the BM samples, applying EPA method 9214. The titration was done afterward by a Metrohm 888 Titrando titrator. The rest of the elements were measured by inductively coupled plasma optical emission spectroscopy (ThermoScientific iCAP 7200) after digestion by aqua regia at 100 °C. The BM particle size distribution (PSD) was determined before and after mechanical activation by using a Retzsch Camsizer X2. Because the upper limit for this equipment was 3 mm, a sieving step was added, samples were sifted with two sieves with square hole sizes of 2.36 and 3.35 mm, and materials with particle sizes of less than 2.36 mm were analyzed with a Retzsch Camsizer X2. The phase identification was done by a PANalytical Empyrean X-ray diffractometer in θ–θ geometry with Co Kα radiation, a beam current of 40 mA, and a beam voltage of 40 kV. The X-ray diffraction (XRD) pattern was measured in the 2θ range of 10–110° with a step size of 0.026°/s. A curved graphite crystal monochromator mounted before a PIXcel 3D detector was used to take care of the fluorescence due to the presence of Fe in the samples. The measured data were evaluated for phase identification using *HighScore Plus* software (version 4.7, PANalytical BV, Almelo, The Netherlands). Additionally, in order to identify the developed phases upon heating, the same X-ray diffractometer (occupied by Cu Kα radiation, a beam current of 40 mA, and voltage of 45 kV), together with an Anton Paar HTK 1200N high-temperature chamber, was utilized to heat up the sample to 1000 °C at a heating rate of 10 °C/min and a He flow rate of 10 mL/min. To analyze the sample at desired temperatures, the sample was held at the set temperature for 1 min before acquiring the XRD pattern, which was measured in the 2θ range of 15–65° with a step size of 0.083°/s. A Zeiss Merlin field-emission-gun scanning electron microscope (SEM) equipped with an Oxford Instruments X-Max energy-dispersive X-ray spectrometer (EDS) detector was employed to morphologically study the samples after the reduction trials.

### 2.4 Thermodynamic Modeling

The thermodynamic studies of the high-temperature reaction were done by *FactSage 8.0* software (Bale et al., 2016). The *Equilib* module was used, and the FactPS, FToxid, and FTmisc databases were selected. The ideal gas and pure solids, besides a series of solution phases (i.e., FToxid-SLAGA, FToxid-SPINA, FToxid-MeO_A, FToxid-NAShB, FToxid-NASlB, and FTmisc-FeLQ), were set as the possible species.

## 3 Results and Discussion

### 3.1 Elemental Analysis

The chemical compositions of HiCo BM and LoCo BM are listed in Table 1. The amount of reducible O was measured at 1070 °C.

**Table 1:** Chemical Composition of HiCo BM and LoCo BM

	element (wt %)				
	Co	Ni	Mn	Li	Cu
HiCo BM	18.7 ± 0.3	5.5 ± 0.1	3.3 ± 0.1	3.6 ± 0.0	5.1 ± 0.1
LoCo BM	4.9 ± 0.1	7.8 ± 0.2	4.5 ± 0.3	2.2 ± 0.1	8.4 ± 0.4

	element (wt %)				
	Al	Fe	C	F	reducible O
HiCo BM	1.5 ± 0.1	0.6 ± 0.0	35.1 ± 0.1	2.5 ± 0.1	17.1 ± 0.3
LoCo BM	5.8 ± 0.4	1.3 ± 0.1	37.5 ± 0.2	2.1 ± 0.1	10.8 ± 0.2

### 3.2 Mechanical Activation

Mechanical activation aims to create defects in the crystalline structure by applying mechanical forces like high friction, collision, and shear.^51^ This method can reduce the particle size, which affects the kinetics of the reactions. The effect of mechanical activation on the reduction of different materials has been studied previously, showing that the reduction rate increases with milling.^45,47,52^ In the latest work done by the authors,^49^ milling of BM resulted in a higher reduction rate. In that study, milling was done for periods of 1, 3, and 5 h. 1 h of milling effectively decreased the particle size and increased the reduction rate, while longer milling periods had a negligible effect. Therefore, this study investigates the effect of milling on the PSD and reduction rates over periods of 30, 60, and 90 min.

#### 3.2.1 PSD

**Figure 1:**
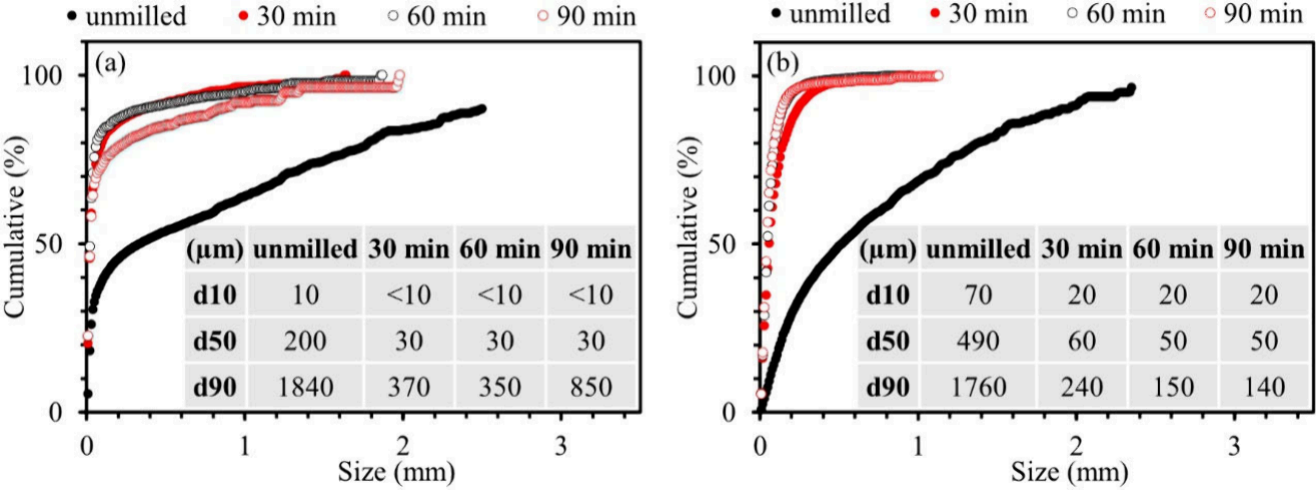
Effect of milling on the PSDs of (a) HiCo BM and (b) LoCo BM (with particle sizes of less than 2.36 mm).

The PSDs of HiCo BM and LoCo BM (with particle sizes of less than 2.36 mm) before and after milling for 30, 60, and 90 min are presented in Figure 1. Manual sieving for particles larger than 2.36 mm showed that 2.5% of HiCo BM and 0.5% of LoCo BM have a particle size of 2.36–3.35 mm and 2.5% of HiCo BM and 1.5% of LoCo BM have a particle size of more than 3.35 mm. Figure 1a shows that *d*_10_, *d*_50_, and *d*_90_ of HiCo BM before milling are 10, 200, and 1840 μm, respectively. These *d* values decrease with milling for 30 and 60 min, but after 90 min of milling, an increase is shown. The effects of different milling periods on d_10_ and d_50_ are the same. For d_90_, milling for 30 and 60 min has almost the same effect, which decreases the d_90_ by approximately 80%. By extra milling (90 min), *d*_90_ increases to 850 μm. For LoCo BM, increasing the milling time results in a decrease in the particle size, as shown in Figure 1b. The main effect occurs after 30 min of milling, where *d*_10_, *d*_50_, and *d*_90_ decrease by 71%, 88%, and 86%, respectively. Milling for longer periods does not affect *d*_10_ but decreases *d*_50_ by 17% and decreases *d*_90_ by 38%.

Generally, in HiCo BM, 30 and 60 min of milling have approximately the same effect, while an increase in the particle size was measured after 90 min of milling. The measured increase in the particle size might be caused by agglomeration of the particles after their sizes pass a threshold^45,53,54^ and hence might not be a true increase in the particle size. For LoCo BM, the main effect on the particle size is observed after 30 min of milling, and the effect of milling for 60 and 90 min is insignificant compared to that of 30 min of milling. The difference in the susceptibility of HiCo and LoCo BM to milling can arise from the intrinsic mechanical properties of the material such as hardness and elasticity module, which can differ in LIB cells with different compositions.^55^

Apart from the effect of mechanical activation on the particle size, it is known that it can also affect the energy state of particles.^51,56^ Changes in the energy state may lead to changes in the kinetics of the reaction occurring at high temperatures, which will be discussed in the next section.

#### 3.2.2 Thermal Analysis

**Figure 2:**
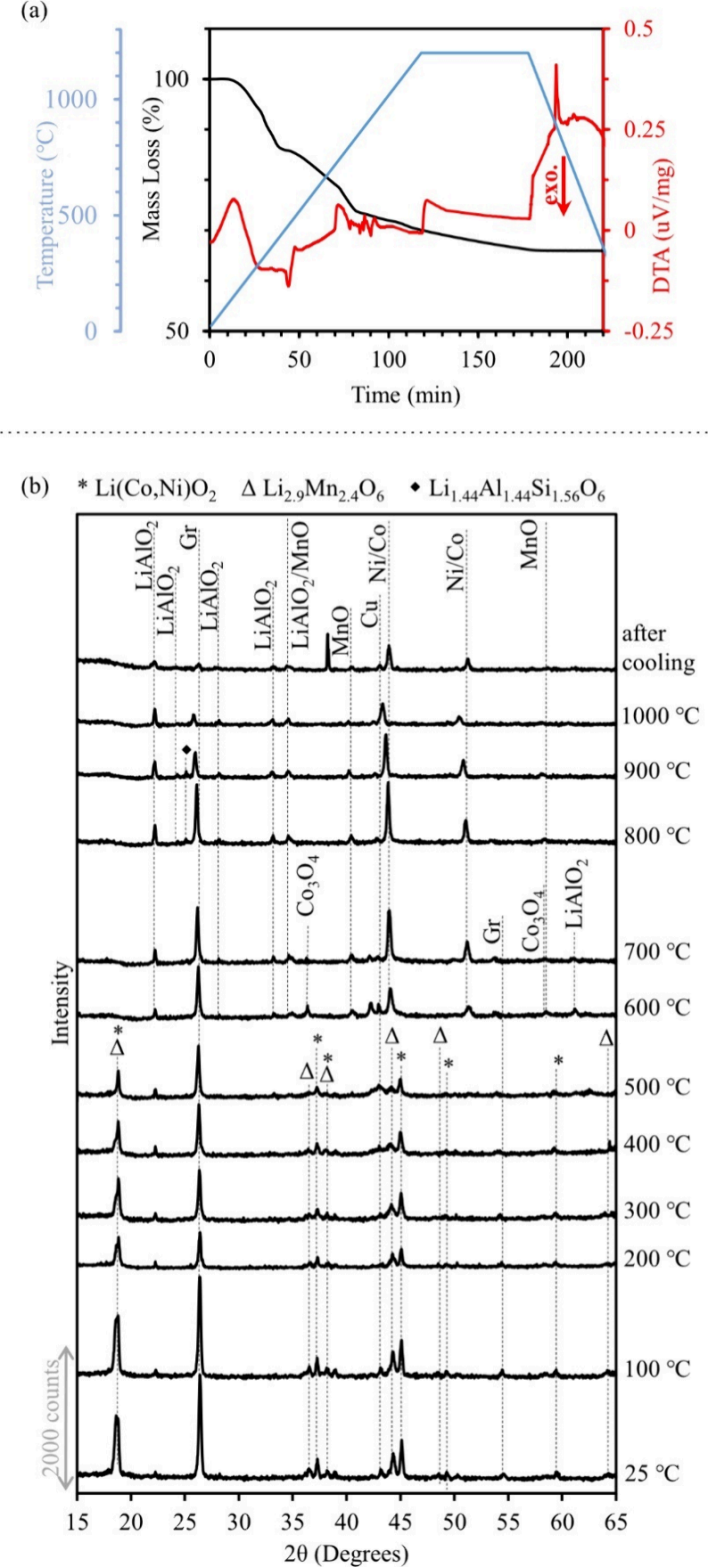
High-temperature transformation of HiCo BM: (a) TGA/DTA graph up to 1200 °C; (b) high-temperature XRD patterns up to 1000 °C.

The results of TGA/DTA and XRD of HiCo BM are presented in Figure 2. These analyses were performed on BM milled for 30 min in order to ensure a homogeneous sample. In the HiCo BM, mass loss begins at approximately 200 °C. The mass loss rate changes at 500–600 °C, which is synchronized with an exothermic reaction. Afterward, the mass loss rate changes at approximately 800 °C during an endothermic reaction. After this, fluctuations can be observed in the DTA curve until 1000 °C, associated with a mass loss at a different rate. When the temperature is increased to 1200 °C and maintained, the mass loss continues at a constant rate. High-temperature XRD profiles demonstrate that Li(Ni,Mn,Co)O_2_, Li_2.9_Mn_2.4_O_6_, graphite, and Cu (anode current collector) are the main phases in HiCo BM up to 500 °C. At 600 °C, the LiMeO_2_ peaks disappear, and Co_3_O_4_, MnO, Ni/Co, and LiAlO_2_ form besides graphite and remain up to 700 °C. At 800 °C, Co_3_O_4_ peaks disappear and a Li-containing compound (Li_1.44_Al_1.44_Si_1.56_O_6_) forms and remains until it disappears at 1000 °C. In addition, a shift in the position of the peaks is observed initially at 800 °C followed by greater shifts at 900 and 1000 °C. After cooling, the pattern shows LiAlO_2_, MnO, Cu, Ni, Co, and graphite as the main phases in the material.

TGA/DTA and high-temperature XRD results from heating LoCo BM up to a temperature of 1200 °C can be found in Figure A1. Similar to the case for HiCo BM, mass loss for LoCo BM begins at approximately 200 °C and continues at nearly the same rate up to 400 °C, synchronized with an endothermic peak. As the temperature increases to approximately 600 °C, the mass loss rate changes, synchronized with an exothermic reaction in the DTA graph. Afterward, the mass loss continues smoothly until it ends at 1200 °C. Diffractograms of LoCo BM during heating show that the main phases are Li(Ni,Mn,Co)O_2_, graphite, and Cu. This combination of phases remains up to 400 °C. At 500 °C, Li(Ni,Mn,Co)O_2_ is replaced by LiMn_2_O_4_, Ni oxide, and Ni/Co. Ni oxides are completely reduced, and MnO forms at 600 °C. At 700 °C, LiAlO_2_ begins to form, and its peaks become stronger at higher temperatures.

The results from these two types of BM indicate that the first step of mass loss (200–500 °C) can be attributed to the decomposition of volatile matters such as binders, solvents, organic materials, and residual electrolyte, with all of the primary phases remaining intact according to the XRD results. The transformation of LiMeO_2_ to its components (Co oxide, Ni, Co, and Li oxide) is completed at 600 °C, although depending on the type of cathode active material, the transformation can begin at lower temperatures (e.g., approximately 400 °C in LoCo BM). This transformation is followed by a reduction of the constituent metal oxides. Moreover, the fluctuations in the DTA curve (Figure 2a) in the temperature range of 800–1000 °C can be related to the formation of an intermediate phase, Li_1.44_Al_1.44_Si_1.56_O_6_, as demonstrated in Figure 2b. Another point to mention is that, although XRD patterns do not show any further reduction after 800 °C, mass loss continues in the BM up to 1200 °C, the highest temperature reached in this study. Possible sources for the continuation of mass loss (based on the thermodynamic modeling and literature survey) include the following: (i) late devolatilization of organics in the BM;^48^ (ii) carbothermic reduction of MnO to metallic Mn;^57^ (iii) reaction of Li/Mn oxides with F, producing Li/Mn fluorides and O [based on thermodynamic modeling, with increasing temperature, MnO(MeO) decreases and Mn(FeLQ), MnO(slag), and MnF_2_(slag) form];^31,58,59^ (iv) reaction of produced O with the existing C, leading to CO production.

To investigate the effect of milling on the kinetics of the reactions and thermal behavior of BM, TGA was conducted on HiCo BM and LoCo BM before milling and after milling for 30, 60, and 90 min, the results of which are presented in Figure A2. Mechanical activation does not have a significant effect on the reduction kinetics of HiCo BM. In LoCo BM, a small increase in the reduction rate is observed in the early stages (200–500 °C), which is related to the decomposition of binders and acetylene black in the BM.^48^ Based on the high-temperature XRD results, this increase cannot be correlated with any reduction processes. Determining the reason for this effect requires further investigation, which is not the focus of the current study.

XRD measurements on the reduced samples after different milling times were conducted to determine the effect of milling on the final products (Figure A3). Generally, the pattern is not affected by the milling. Similar to the high-temperature XRD patterns (the sample cooled after heating at 1000 °C), the main phases in both types of reduced BM are LiAlO_2_, graphite, Ni/Co/Cu, and MnO. This shows the following: (i) Co and Ni oxides are completely reduced; (ii) MnO does not reduce (or at least not a complete reduction) under the current experimental conditions; (iii) although part of the graphite is consumed during the reduction of existing metal oxides, graphite is still present in the BM; (iv) residual Al from the cathode current collectors react with Li in the cathode active material and form LiAlO_2_; (v) mechanical activation does not affect the final products of BM reduction.

Li distribution at different temperatures was modeled using *FactSage*, as presented in Figure A4. The results reveal that the amount of Al significantly influences the destination of Li. In HiCo BM, depending on the temperature, Li_2_CO_3_ and Li_2_O are the most abundant Li-containing compounds, whereas in LoCo BM with a higher Al content, Li is mainly found in the form of LiAlO_2_. The formation of LiF and LiAlF_4_ is also thermodynamically possible, which hinders F evaporation. It is also notable that *FactSage* does not predict Li in the gas phase. When these thermodynamic data are combined with the results from high-temperature XRD patterns, it can be perceived that LiAlO_2_ starts to form after a temperature of 600 °C is reached. At lower temperatures, it is thermodynamically more likely to find Li in other forms, e.g., Li_2_O, Li_2_CO_3_, and LiF. Thus, it can be concluded that LiAlO_2_ is the product of the heating of BM at 1200 °C. Additionally, although the off-gas has not been analyzed, thermodynamic calculations do not indicate Li evaporation.

### 3.3 Production of Fe-Based Alloys

The amount of graphite in the BM exceeds what is required for the reduction of LiMeO_2_, as demonstrated in Figure A3. In the current work, the method used to consume the remaining graphite was to add a metal oxide (hematite in this study) to the BM. The extra graphite was utilized as a reducing agent to produce an Fe-based alloy containing valuable reducible metals in the BM as alloying elements.

**Figure 3:**
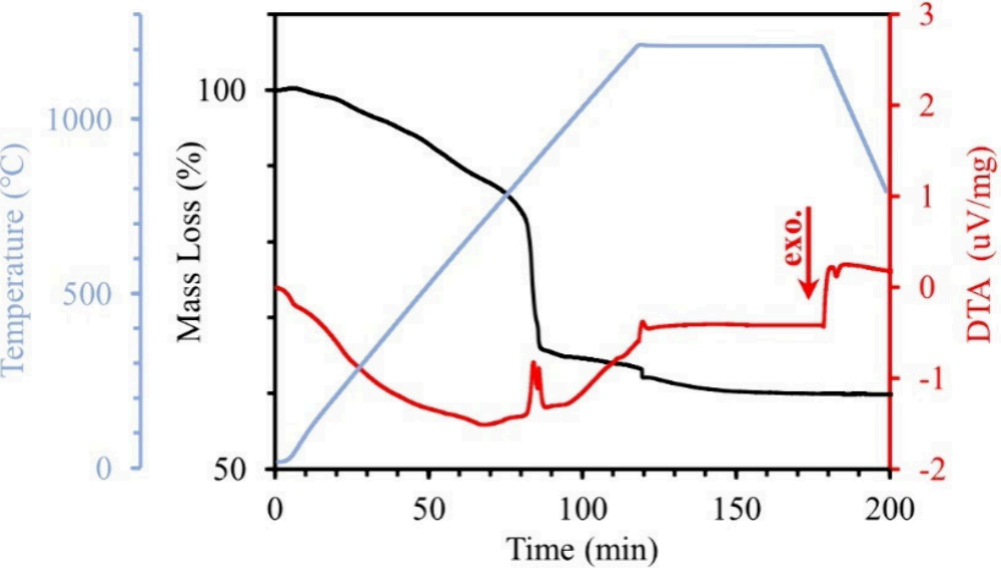
TGA/DTA graph of a mixture of hematite with HiCo BM, heat treated from room temperature to 1200 °C.

Figure 3 presents the TGA and DTA results obtained during heating of the hematite–BM mixture (in this section, the results addressing LoCo BM can be found in Figures A5–A7). Mass loss begins at 100–200 °C and continues with an almost constant slope until approximately 400 °C in HiCo BM and 500 °C in LoCo BM. At approximately 700 °C, the mass loss slope changes, followed by an endothermic reaction at 800–900 °C, which accompanies a steep mass loss.

**Figure 4:**
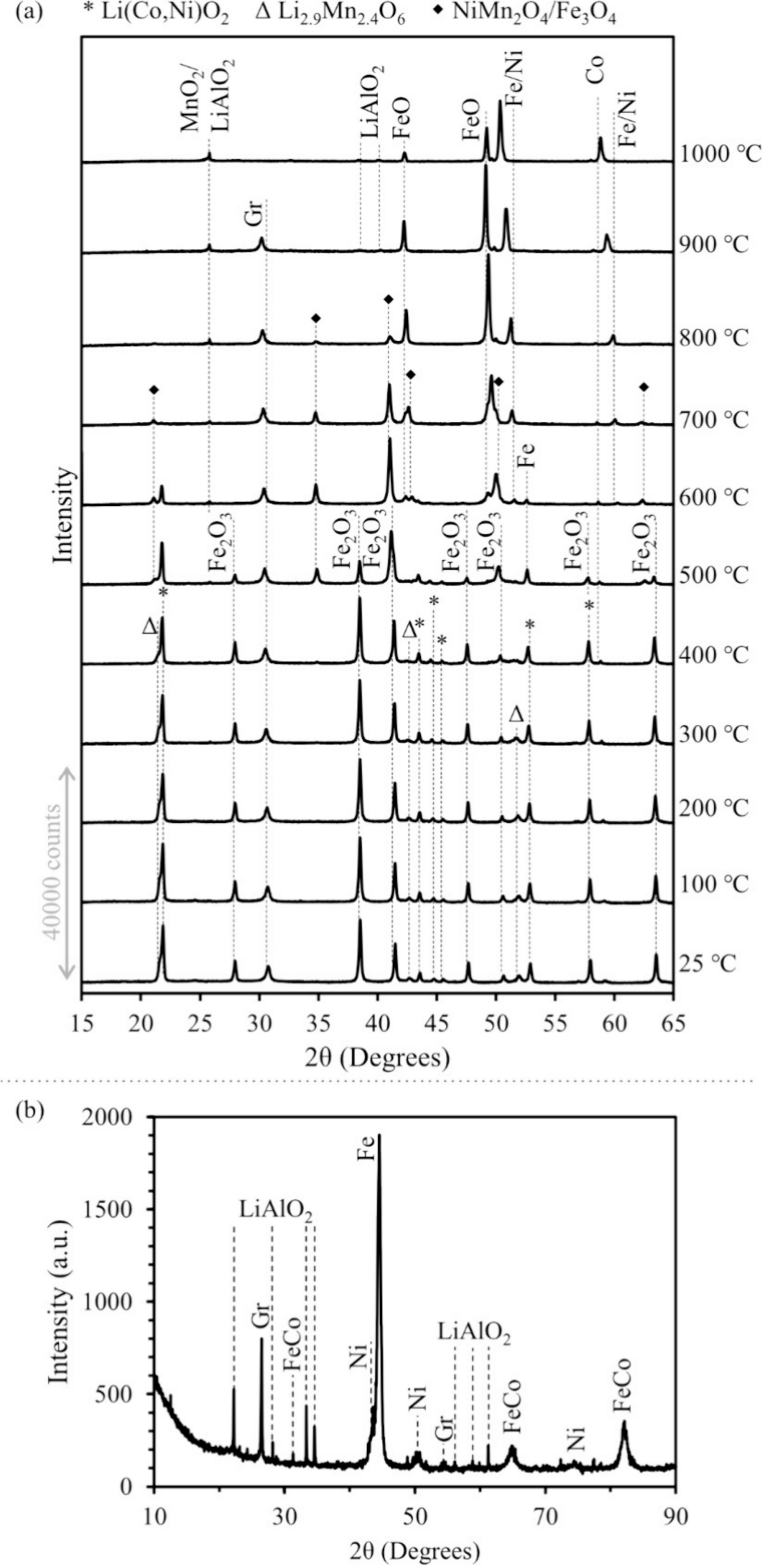
XRD patterns of the mixture of hematite and HiCo BM: (a) during heating; (b) after reduction at 1200 °C.

High-temperature XRD in Figure 4a shows that LiMeO_2_ remains intact until 400 °C and hematite until 500 °C. At these temperatures, LiMeO_2_ transforms to LiAlO_2_ and NiMn_2_O_4_, and hematite reduces to Fe_3_O_4_. These new phases persist until 700–800 °C, after which Co, Ni, Fe, and FeO peaks appear in the pattern. The graphite peak could be detected up to 900 °C.

Figure 4b presents the formed phases after reduction at 1200 °C, as determined by XRD. The results show that Fe, Co, and Ni oxides are completely reduced to their corresponding metallic phases and Li is present in the form of LiAlO_2_. There are also some weak graphite peaks in the pattern, addressing the hematite–HiCo BM mixture. The remaining C in the sample after reduction was measured, which were 8.8 and 2.0 wt % in the mixture of hematite with HiCo and LoCo BM, respectively.

**Figure 5:**
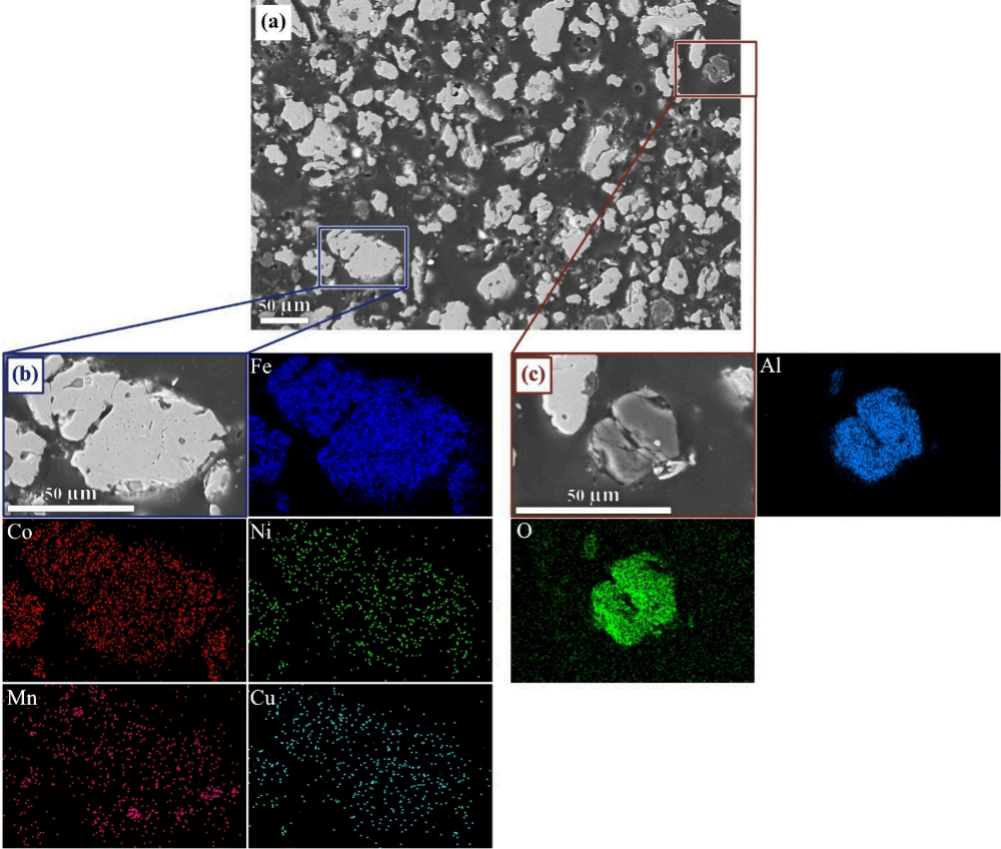
(a) BSE image of the hematite and HiCo BM mixture after reduction at 1200 °C. EDS maps of (b) a metallic particle and (c) a nonmetallic particle.

The backscattered electron (BSE) images of the reduced mixtures, obtained by SEM–EDS, are presented in Figure 5 (the image of the reduced hematite–LoCo BM mixture can be found in Figure A7). The morphology of the particles shows that reduction occurred in the solid state, and metallic particles containing Fe, Co, Ni, and traces of Mn are observed. The low-mass particles appear to contain Al and O. This observation aligns with the XRD results, confirming the reduction of hematite. The particles containing Al and O likely represent LiAlO_2_, the phase identified with XRD, although Li is not detectable by EDS.

When the results are combined, it can be stated that the preliminary mass loss may be attributed to the evaporation of volatile matter in the BM. Upon reaching 400–600 °C, the cathode active material transforms into its constituents, including LiAlO_2_, Co, and NiMn_2_O_4_. Hematite reduction initiates between 500 and 600 °C, and the reduction of NiMn_2_O_4_ and Fe_3_O_4_ (to FeO) occurs at 800–900 °C during an endothermic reaction. At 1000 °C, FeO is still present in the sample, while the XRD pattern after cooling from 1200 °C shows the complete reduction of FeO to Fe. As revealed by SEM–EDS investigation, an alloy is formed during the reduction trial. The morphology of the particles shows that reactions predominantly occurred in the solid state.

Thermodynamic calculation conducted using *FactSage* (Figure A8) showed that hematite, along with the metal oxides present in the BM, undergoes reduction and forms a Fe-based alloy containing Co, Ni, Mn, Cu, and C. This indicates that most of Li does not evaporate and remains in the sample either in the slag or in the form of solid LiAlO_2_. When the results from HiCo BM are compared to those of LoCo BM, it can be stated that if the Co content exceeds the solubility limit of Fe, as observed in the HiCo BM, it leads to the formation of Co in the solid state.

In this study, a pyrometallurgical approach for recycling LIBs and in situ alloy-making has been investigated. BM was obtained from a mixture of different LIB types, similar to the input materials in recycling facilities. When the results obtained from the current and previous studies by the authors are combined,^48,49^ it can be inferred that 600 °C represents a critical temperature in the heat treatment of BM: (i) complete transformation of LiMeO_2_ to its constituents is expected at 600 °C; (ii) in the presence of Al, LiAlO_2_ begins to form at temperatures higher than 600 °C. Moreover, the temperature of 800 °C is the point of complete reduction of Co and Ni oxides, while LiAlO_2_ and part of MnO persist up to 1200 °C.

Another subject of focus was the feasibility of in situ alloy-making by incorporating a metal oxide (hematite) into the BM. A temperature of 1200 °C was chosen for the reduction trials, during which complete reduction occurred in the solid state. It was observed that it is still feasible to obtain an Fe–Co–Ni alloy at this temperature. Observing the high-temperature transformation of a mixture of hematite and BM revealed that, in addition to BM transformation, hematite reduction began at approximately 500 °C, leading to the formation of Fe_3_O_4_. The FeO phase was detected at 600 °C, coexisting with Fe_3_O_4_ until 700 °C. FeO persisted alongside Fe up to 1000 °C, while it completely reduced to Fe after reaching a temperature of 1200 °C. Furthermore, thermodynamics showed that nearly all Li forms LiAlO_2_ and does not evaporate. In situ alloy-making was seen as a potential method for utilizing BM, even though BM contains some other elements that cannot be dismissed, including Li, F, Mn, and Al. The addition of slag formers to this system can help with cleaning of the steel and separating impurities, which are the focus of the next step of this study.

The effect of mechanical activation on the BM thermal behavior is an area of interest that the authors studied in their previous work, focusing on LCO and NMC111 BMs, which demonstrated an enhancement in the kinetics of the reduction reaction.^49^ In the current study, the same approach (with some changes in the milling time) was employed for the mixed BM, in which no significant effect was observed. This difference may arise from variations in the techniques for producing BM; i.e., if high-energy mechanical forces were already employed during BM production, further mechanical activation might appear to be ineffective. Another factor to consider is variation in the materials. If the kinetics of a reaction is already high, additional mechanical activation may not improve the reaction rate. From this, one can conclude that mechanical activation does not necessarily affect the kinetics of the reduction reaction, depending on the history of the BM.

## 4 Conclusion

The high-temperature behavior of BM obtained from two mixtures of different NMC LIBs, with high and low Co contents, was investigated. A temperature of 600 °C is deemed appropriate for complete transformation of the cathode active material to its constituents. Based on high-temperature phase identification and thermodynamic calculations, if Al is present, LiAlO_2_ forms at temperatures higher than 600 °C, while Li_2_CO_3_ is the predominant Li-containing phase at 600 °C. Consequently, depending on the desired input material for the subsequent Li purification step, the temperature for the heat treatment can be chosen accordingly. Additionally, 800 °C is the temperature suitable for obtaining metallic Co and Ni. Moreover, LiAlO_2_ and part of MnO remain unreduced after the heating trial at a maximum temperature of 1200 °C.

The feasibility of in situ alloy-making, by introducing external metal oxides (hematite) to the BM, has also been considered. It was observed that, upon reaching 1200 °C, during a solid-state reduction reaction, an Fe-based alloy is formed, containing the valuable metallic elements in the BM, including Co and Ni. Furthermore, thermodynamic modeling indicates that Li forms LiAlO_2_ and does not evaporate.

In addition, the impact of mechanical activation on the reduction kinetics of the BM was investigated. It was concluded that mechanical activation does not necessarily affect the thermal behavior of the BM.

## Supporting Information Available

Complementary results from experiments and thermodynamic modeling (PDF)
