# Supplementary material for: Pyrometallurgical Approach to Extracting Valuable Metals from a Combination of Diverse Li-Ion Batteries’ Black Mass
Source: ACS Sustain Resour Manag. 2024 Jul 22;1(8):1759–67. doi: 10.1021/acssusresmgt.4c00117 (PMC11345943; doi:10.1021/acssusresmgt.4c00117)
Supplement: Supplementary file 1 — rm4c00117_si_001.pdf [file rm4c00117_si_001.pdf]

## Title Page

| | |
|---|---|
| **Manuscript title** | A Pyrometallurgical Approach to Extract Valuable Metals from a Combination of Diverse Li-ion Batteries' Black Mass |
| **Author names and affiliations** | Safoura Babanejad[1]*, Hesham Ahmed[1,2], Charlotte Andersson[1], Elsayed Mousa[2,3]<br><br>[1] Department of Civil, Environmental and Natural Resource Engineering, Process Metallurgy, Minerals and Metallurgical Engineering, Luleå University of Technology, 971 87 Luleå, Sweden<br>[2] Central Metallurgical Research and Development Institute, P.O. Box 87, Helwan 114 21, Egypt<br>[3] SWERIM AB, Aronstorpsvägen 1, 974 37, Luleå, Sweden |
| **Corresponding author** | Safoura Babanejad<br><br>safbab@ltu.se |

**Appendix A, including 6 pages: Figs. A1-A8**

# Supporting information

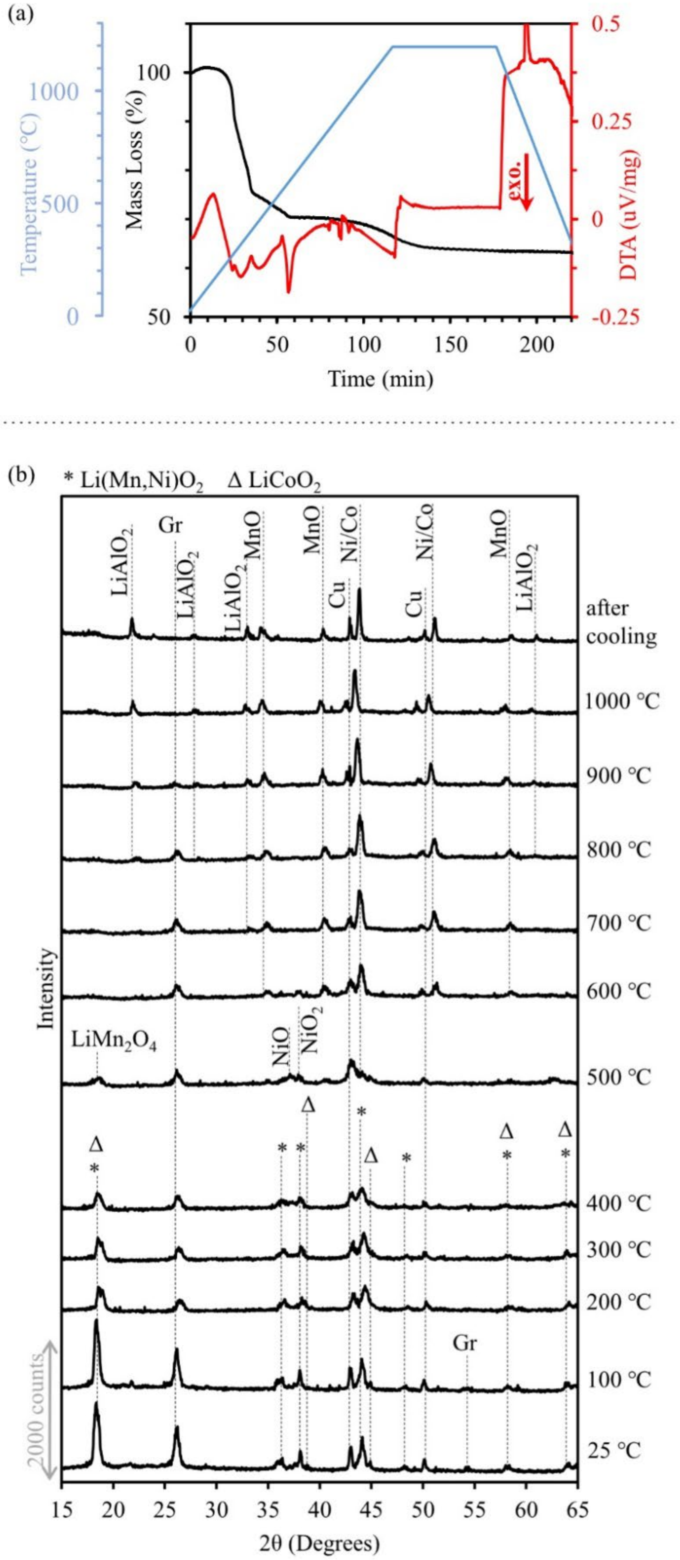


*Fig. A1. High-temperature transformation of LoCo BM: (a) TGA/DTA graph up to 1200 °C, and (b) high-temperature XRD patterns up to 1000 °C.*

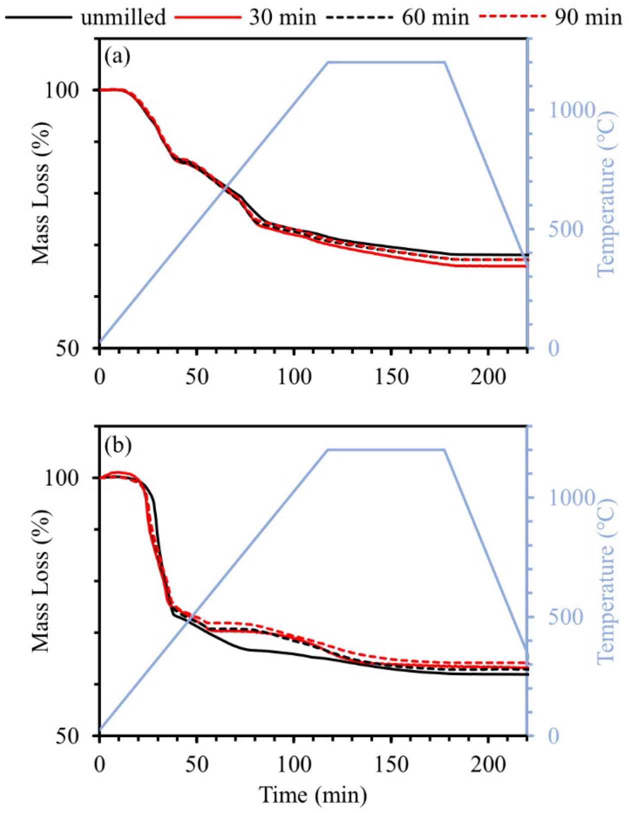


*Fig. A2. Effect of mechanical activation on the thermal behavior of (a) HiCo BM, and (b) LoCo BM.*

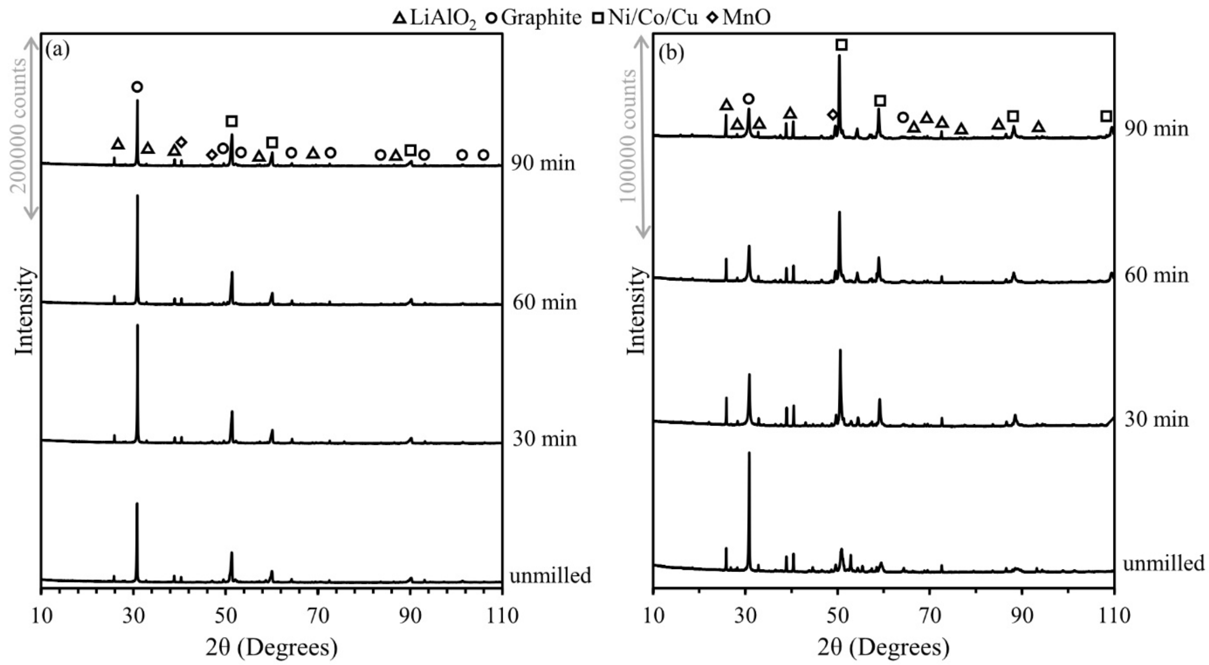


*Fig. A3. XRD patterns of (a) HiCo BM and (b) LoCo BM (unmilled and mechanically activated) after reduction.*

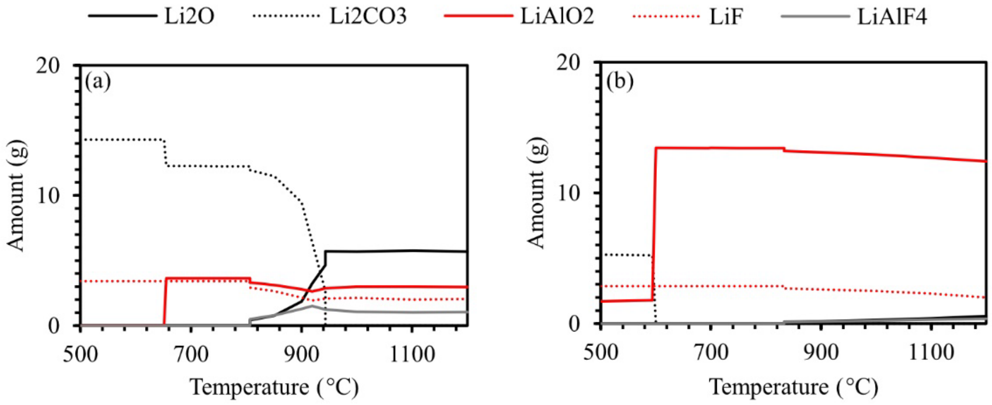


*Fig. A4. Phase distribution of Li during heating (a) HiCo BM and (b) LoCo BM, modelled by FactSage (all phases in the solid state).*

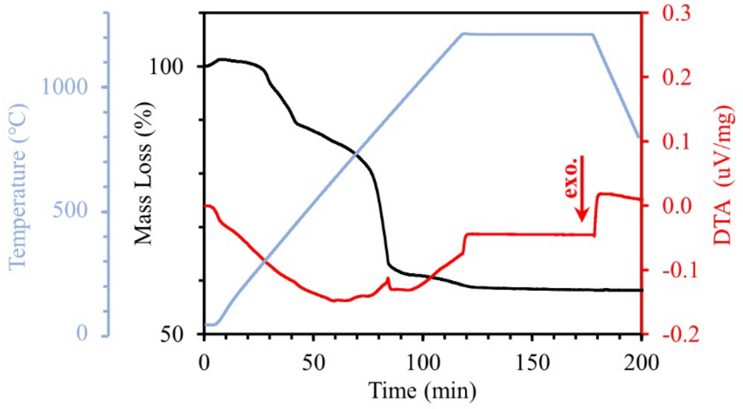


*Fig. A5. TGA/DTA graph of the mixture of hematite with LoCo BM, heat treated from room temperature to 1200 °C.*

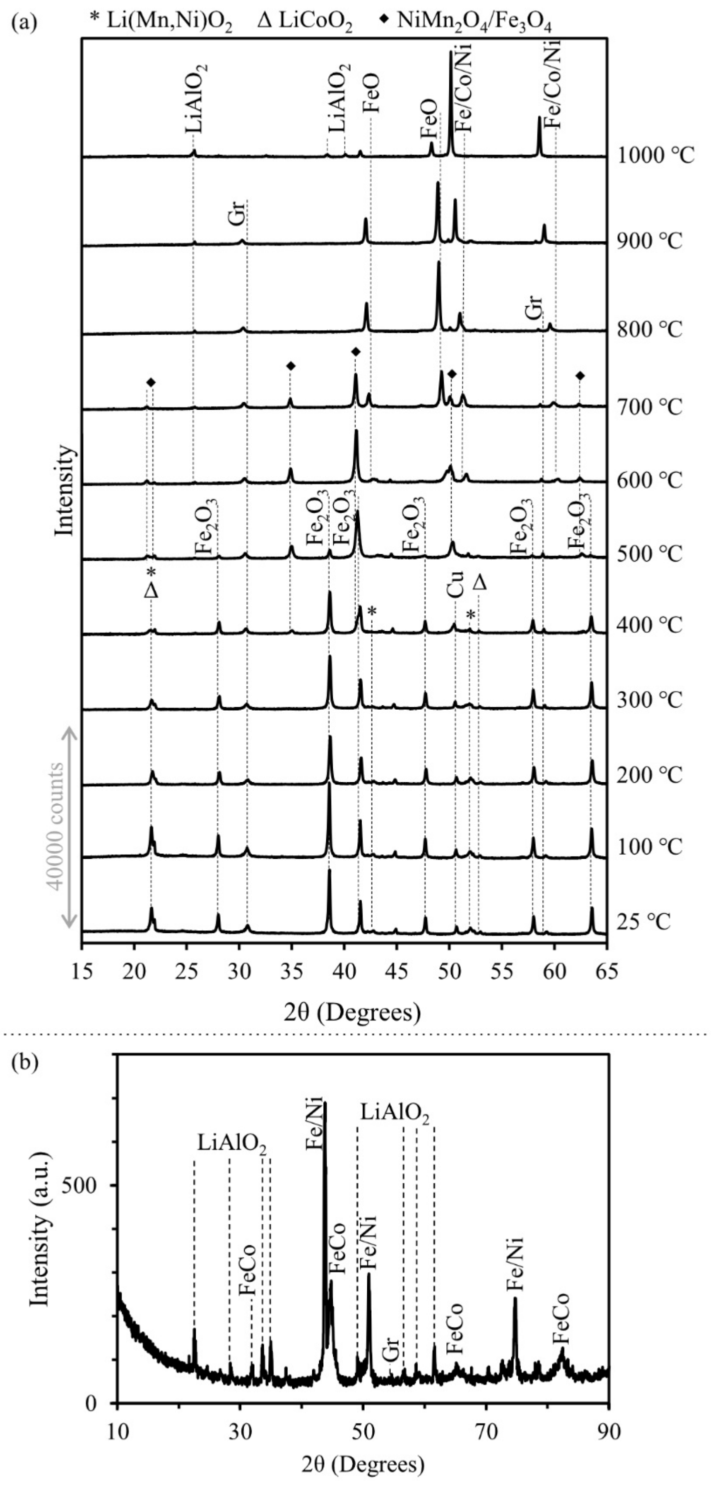


*Fig. A6. XRD patterns of the mixture of hematite and LoCo BM: (a) during heating and (b) after reduction at 1200 °C.*

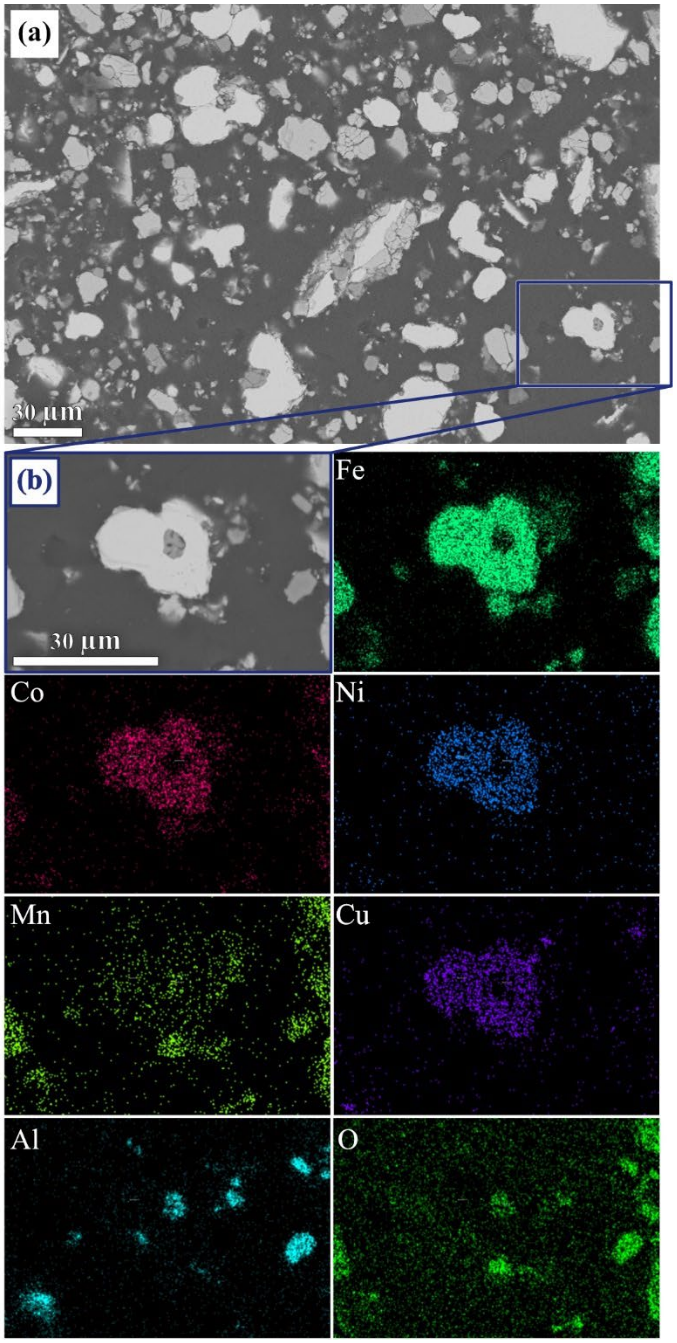


*Fig. A7. (a) BSE image and (b) EDS map of hematite and LoCo BM mixture after reduction at 1200 °C*

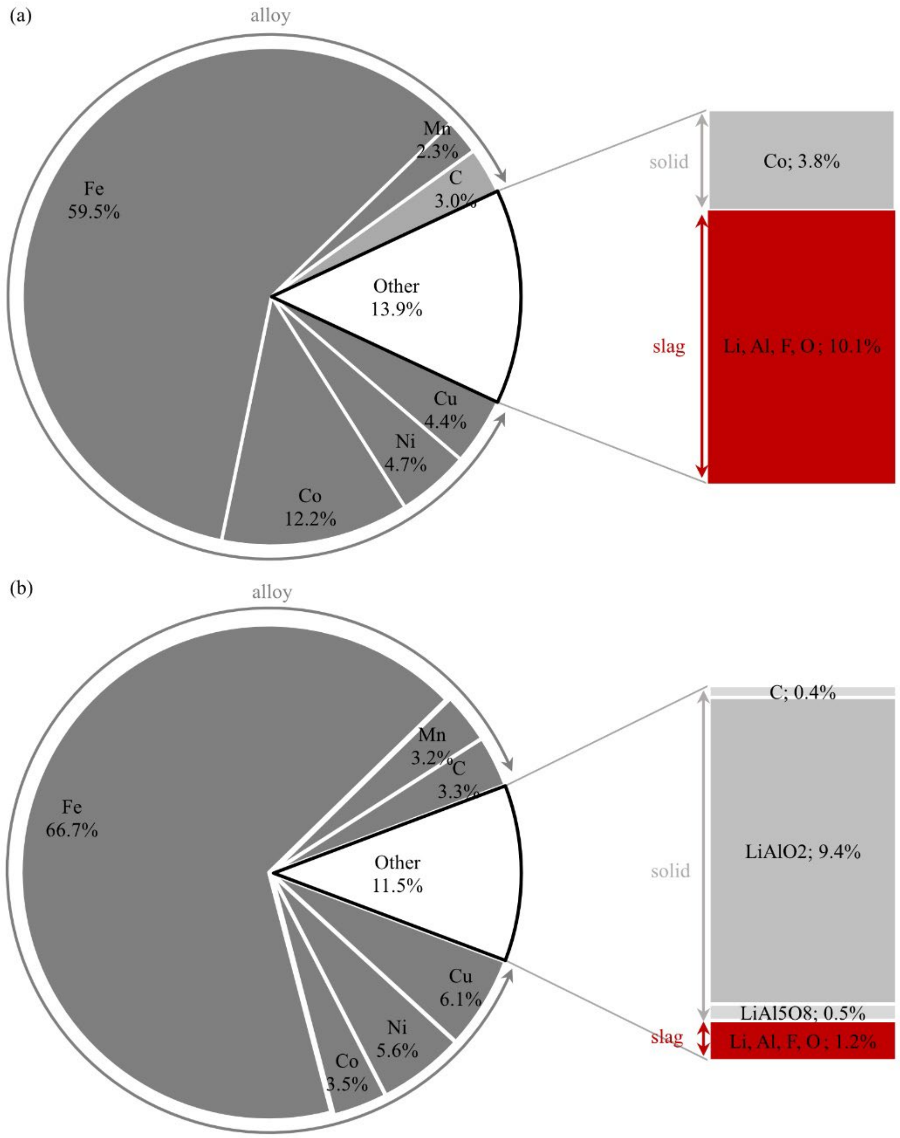


*Fig. A8. Thermodynamic calculations by FactSage: Reduction of hematite mixture with (a) HiCo BM and (b) LoCo BM, at 1200 °C.*
